# The role of higher education and civic involvement in converting young adults' social responsibility to prosocial behavior

**DOI:** 10.1038/s41598-023-29562-4

**Published:** 2023-02-13

**Authors:** Nikša Alfirević, Maja Arslanagić-Kalajdžić, Žan Lep

**Affiliations:** 1 University of Split, Split, Croatia; 2 University of Sarajevo, Sarajevo, Bosnia and Herzegovina; 3 University of Ljubljana, Ljubljana, Slovenia; 4 Educational Research Institute, Ljubljana, Slovenia

**Keywords:** Psychology and behaviour, Socioeconomic scenarios

## Abstract

This study investigates the indirect mechanisms relevant to converting young adults' prosocial attitudes and individual responsibility into their prosocial behavior. Our results are based on a sample of 530 young adults studying at three public regional business schools in South East Europe. They show a significant favorable influence on young adults' civic and political involvement, mediating the relationship between individual responsibility attitudes and prosocial behavior. However, this would not have been expected based on previous research. Another indirect path between the same variables is modeled using a hypothesized moderated mediation effect. The institutional influence of higher education proves to be a significant mediator of the proposed relationship, moderated by the amount of educational content in the fields of ethics, social and environmental responsibility. At mid-and-high levels of exposure to relevant educational content, this indirect path significantly influences the developing young adults' pro-environmental behaviors. The study results are discussed from the viewpoint of peripheral regions with a history of dysfunctional social capital mechanisms.

## Introduction

As new students and young professionals belonging to 'Generation Z' (born between 1995 and 2015) start filling managerial roles, their values, ethical and social corporate responsibility perceptions are becoming the determining factor for the socially responsible behavior of organizations^1^. This suggests examining how young adults' attitudes are converted into actual behaviors, including the mechanisms, to provide indirect support to such a process. With a clear understanding of behavior formulation at the individual level, it will be easier to understand and predict similar processes in organizational and other social settings.

Young adults experience the earliest stages of their professional socialization by participating in the education process, especially at the higher education level^2^. Being central to the socialization process, we analyze the potential influence of the higher education institutions (HEIs) at which they study to support prosocial behaviors. Prosocial behaviors are generally defined as voluntary behaviors aimed at helping others^3^. They can be as direct as helping a person in need or as broad as volunteering for the (perceived) benefit of society at large. In the psychological literature, prosocial behaviors can be operationalized by considering their underlying mechanisms, for example, motivation to help, perceived norms, internalized prosocial value orientations, moral reasoning, and social competence^4–6^. Thus, the concept of prosocial behavior comprises a broad spectrum of actions such as helping, cooperating, sharing, comforting, empathy, altruism, volunteering, and donating^6–8^, which could be both private and public^9^, or spontaneous or planned^10^.

Regarding socialization, youth prosocial behavior can be fostered in various settings, including educational settings [e.g.,^11,12^]. Following a history of prosocial higher education since the seventeenth century^13^, at least in the Anglo-Saxon context, there is also evidence of the general prosocial impact of higher education^14^. However, this seems to vary with the field of study^15^, depending on the professional values.

Dependence of the mechanism on the professional context involves the potential influence of individual self-selection based on the perceived compatibility between individual and professional values. This is especially relevant for business schools^16^, which are traditionally supposed to train students to become rational decision-makers, aiming to maximize profits and other organizational benefits. It is unclear if formal business ethics education matters^17,18^. In addition, there is mixed empirical support to the claim that formal ethics education or training can improve the moral reasoning of business students^19–21^ or that the obtained effects are sustainable in the long term^22^. Therefore, we chose the population of undergraduate business students for this study. It seems reasonable, in our opinion, to confront the examination of the empirical results with the most challenging context when generalizing young adults' attitudinal and behavioral patterns.

Young adults' political engagements and culture enhance their prosocial behaviors^23^. At the same time, involvement with civic groups and structured civic activities can promote prosocial behavior^24^, as opposed to peer-oriented leisure activities, without the skill-building orientation^25,26^. Due to the lack of research on young adults' prosocial behavior in the South East European (SEE) region, it is unclear if the dysfunctional patterns of social capital in the regional communities^27^ influence the prosocial outcomes of the educational processes and what might be the direction of this potential relationship. However, given the previously undocumented influence of the Bulgarian and Romanian immigrants on civic engagement in their home countries^28^, we expected to encounter a significant influence of regional contextual factors, which have not been discussed previously.

In this study, we use a sample of 530 undergraduate students of business from two SEE countries (Croatia and Bosnia & Herzegovina) to address the following research questions:

- What is the contribution of higher education institutions (HEIs) and institutionalized ethics to formulating young adults' prosocial behaviors?
- How does political and civic engagement contribute to the formulation of prosocial behaviors?
- Are there any mechanisms at work, which could be related to the specific regional contexts, or circumstances?

Empirical results of this study contribute to understanding the role of less visible mechanisms and contextual factors, which might be at work in small countries and cultures, as related to the formulation of young adults' prosocial behaviors.

## Literature review, hypotheses development, and research model

**Figure 1:**
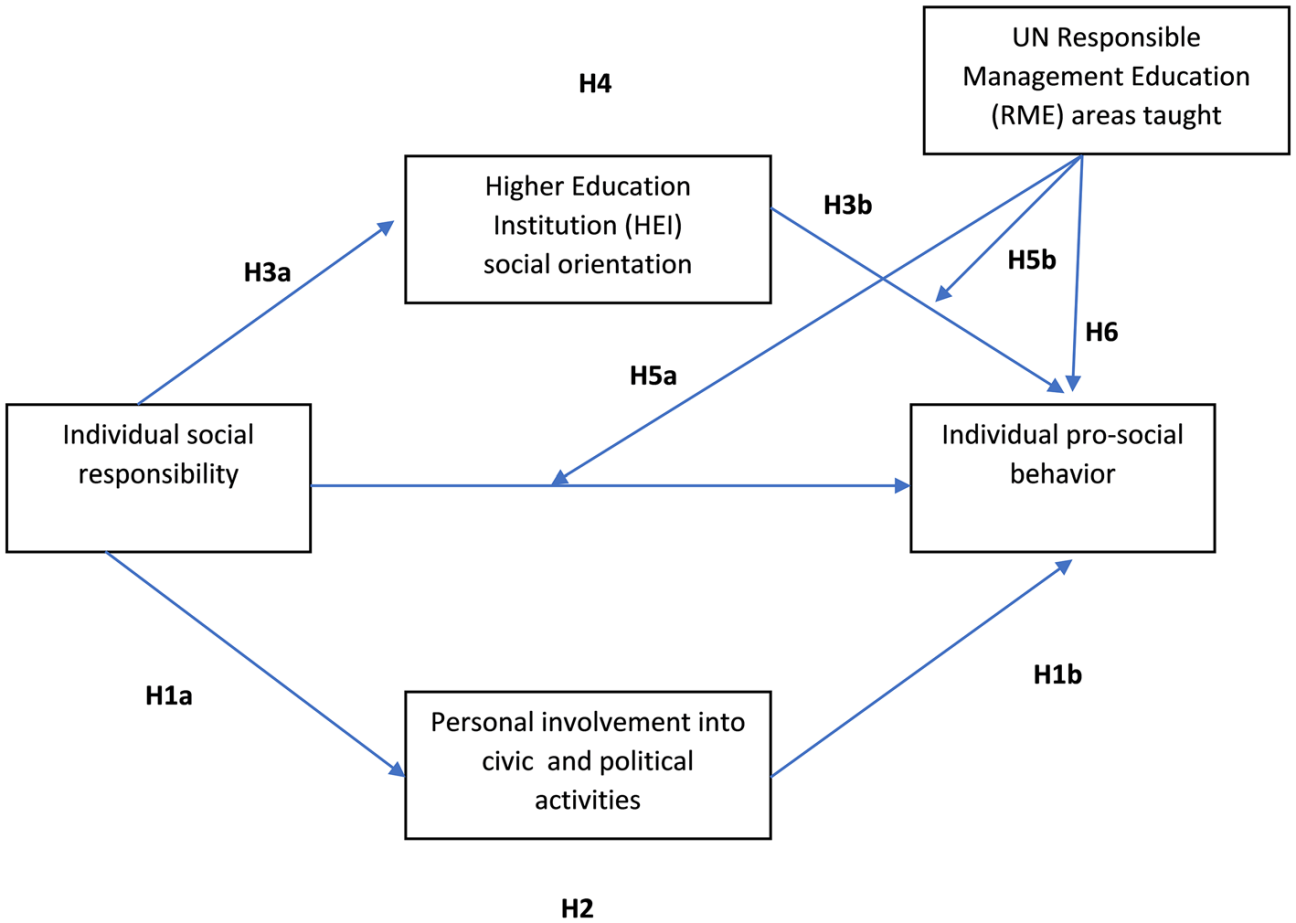
Research model and hypotheses.

Given the study's objectives, we formulated a research model accounting for variables on both personal and institutional level (see Fig. 1). The former includes individual social responsibility, personal involvement in civic and political activities, and individual prosocial behavior. The latter considers the social orientation of a Higher Education Institution (HEI) as perceived by the individual and the extent to which Responsible Management Education (RME) areas are taught. The model is based on the classical cognitive approach to explaining the role of individual and environmental factors in behavior.

The cognitive approach defines perceptions of the environment as a key explanatory factor of individual behavior (referred to in our model as the Higher Education Institution social orientation). Specifically, perceptions are mediating cognitions that characterize the environment's meaning for the individual^29^ and the meaning attributed to the environment from both individual characteristics and characteristics of the environment^30^. The most important individual characteristics that determine the perceptions of the environment are values—they define "what is important to the individual"^31:328^ and create cognitive schemes through which the evaluation of the environment in terms of importance to the individual takes place^30,31^.

Individual characteristics, defined in terms of values that social responsibility has in the individual value system, are represented in Fig. 1 by the constructs of Individual social responsibility and Personal involvement in civic nonprofit and political activities. Psychological climate theory^29^ was extended in this study to include an element of the "objective" environment, which is an essential element in shaping the value system, i.e., higher education, particularly during adolescence and the transition to adulthood^32^. Figure 1 presents a predictive model of prosocial behavior based on the theory of psychological climate^29^ and the general interactionist^30^ approach to predicting individual behavior.

### Individual dispositions of prosocial behavior

Over the last decade, myriad data points to lower rates of youth political participation in the SEE region and beyond^33,34^. The observed (lower) rates, however, do not translate into the disengagement of youth and a lack of their interest in the current topics^35^. Indeed, anecdotal evidence shows significant potential for young people to engage in civic behavior when a specific topic is perceived as essential and when they sense adequate levels of agency. Perhaps the most evident example, observed in recent years, are various environmental movements that attract young people. For some youth, environmental responsibility represents a moral issue, and participation in environmental movements provides them with the opportunity for alternative forms of prosocial engagement that might be more adequate for them.

Such engagement is exceedingly vital as previous participation is one of the strongest predictors of future participation^36^. Prosocial behavior in youth is further influenced by several individual traits and psychological characteristics, such as demographics, socialization in families, peer groups, and schools^12,37^, values^38^, moral reasoning and empathy^39,40^, and autonomous motivation. Our study focuses on individual social responsibility, which comprises personal caring and responsibility stemming from individual moral judgments. Conceptually, this construct is related to self-perceptions of youth, i.e., their civic and moral identity^41^, attitudes, and values^42^ which all contribute to higher participation. Thus, we hypothesized that individual social responsibility would predict higher personal involvement in civic and political activities (H1a), and more frequent prosocial behavior (H1b). Moreover, as different forms of civic behaviors, namely political, civic, prosocial, and pro-environmental^12,43^, are interrelated, we expect that personal involvement in civic and political activities will also mediate the relationship between individual social responsibility and prosocial behavior (H2).

### The role of higher education institutions

Besides considering the well-established relationships between personal dispositions and prosocial behavior, the goal of this study was to explore the contribution of higher education institutions and their social responsibility to the promotion of the prosocial behavior of youth. Educational institutions represent a vital socialization environment for children, adolescents, and emerging adults^2,43^. However, the role of higher education institutions can be critical because of the developmental characteristics of their students and the interaction between students and institutions. Higher education students are among the most active social groups^44^. They experience relatively few external obstacles to participation, while HEIs provide a favorable environment for moral development linked to prosocial behavior^4^. Compared to adolescents, emerging adult students are also generally less restricted by parents and teachers. They have comparably more free time to participate than their seniors who deal with other social obligations—e.g., family and work^37,45^.

Business schools have experienced a growing interest in sustainable development, countering the pervasive lay beliefs about business students being detached from prosocial engagement. While there is some self-selection among business school students^46^, the evidence does not support that they are less prosocial or that business schools socialize them to be more individualistic^15^. On the contrary, business schools were found to change students' attitudes about corporate responsibility^47^. At the same time, the inclusion of responsible management education was linked to higher students' self-transcendence and more positive attitudes toward corporate social responsibility^48^.

Moreover, higher education students are not merely passive receivers of knowledge but are actively involved in shaping the execution of the curricula, for example by expressing interest in topics such as RME^49^, managing the institutions (e.g., participation in student bodies), and organizing extracurricular activities at the institution. In this way, they are also agents of change in the organizational culture—if they, for example, demand changes in how plastic waste is managed, the institution might comply and change its ways. Coupled with the previously described self-selection when enrolling in specific study programs and extant research findings on the role of students' idealism in shaping both their pro-environmental behavior^50^ and HEI sustainability^51^, we expected that individual social responsibility will be linked through bottom-up processes with the social orientation of an HEI (H3a) and that social orientation of an HEI will be linked—via top-down processes—with prosocial behavior of the students (H3b). Such hypothesized processual mechanisms lead to the hypothesis that individual social responsibility and prosocial behavior are mediated by the HEI social orientation construct (H4).

### Academic teaching and learning of Responsible Management Education

The pathways toward prosocial behavior we proposed thus far are all based on the participants' perceptions. While this is important considering the discussed importance of individual attitudes, values, and perceptions, we wanted to integrate both subjective and (somewhat) objective factors in our model. Specifically, we explored the relationship between student-assessed (implicit) HEI social responsibility and explicit messages signaled through official curricula as some previous findings warn of the pitfalls of their misalignment^52^. To assess the explicit level of HEI prosocial orientation, we considered the concept of Responsible Management Education (RME), which proposes teaching business ethics, Corporate Social Responsibility (CSR), and environmental sustainability topics^53^ topics (areas) to students. Specifically, we proposed that the amount of direct teaching in RME areas would moderate the relationship between individual social responsibility (H5a) and prosocial behavior and the relationship between HEI social orientation and individual prosocial behavior (H5b). In addition, we consider the potential direct relationship between the RME areas taught and individual prosocial behavior (H6).

These hypotheses are grounded in the well-established role of knowledge in predicting various forms of civic participation^54,55^. However, we acknowledge that the amount of teaching might not translate directly to students' knowledge. Still, some propose that knowledge could be considered a moderator between psychological variables and civic participation in youth (see^56^ for a review), which we tested in our model. Further, we posit that a higher amount of teaching, if congruent with hidden curricula expressed in the measure of HEI social responsibility, would strengthen the relationship between HEI social responsibility and individual prosocial behavior.

## Methods

### Research sample

This study is based on student responses to a Web-based questionnaire, with the introduction page of the survey asking for students’ informed consent to participate. Participants were informed of the purpose of the study, data collection practices, and the guarantees of anonymity and privacy, including the statistical reporting of the obtained research results. Only students accepting the terms of the informed consent were allowed to fill in the survey.

Relevant institutional guidelines and regulations were followed in all stages of the research process. Compliance has been reviewed and approved by the institutional Ethics committee of the Faculty of Arts at the University of Ljubljana, Slovenia (No. 231-2021). The data collection included undergraduate business and economics students at three mid-sized regional business schools in Croatia and Bosnia and Herzegovina. The entire undergraduate student body was informed of the survey by posting a Web link (URL) on the Learning Management System internal discussion forum. The authors had no means to track the (non)participation of invited students in the survey since personal details, such as names, course registration numbers, and IP addresses, were not collected. Responses to demographic data, including gender, age, study program, average study grades, and social status, were optional.

The sample for this study consists of 530 responses obtained from undergraduate students of business from two countries (Croatia and Bosnia & Herzegovina) located in the South East Europe. The survey was conducted in the academic year 2020/21 using non-random sampling. An invitation posted on the internally available Web pages (i.e., the Moodle Learning Management System pages) has been extended to the entire student body in the 2nd year of the undergraduate business program. In Croatia, data collection has been performed at the Faculty of Economics, Business and Tourism Split, the second largest in the country. Since Bosnia & Herzegovina has a complex administrative structure of two political entities, we collected data at the country’s two largest public business schools (affiliated with the University of Sarajevo and the University of Banja Luka), located in the administrative centers of the two entities.

This study is an initial report of a larger research project, aiming to cover the entire region of SEE and countries from Central East Europe. It is building upon the results of recent empirical research in the same region^1^, which show the existence of regional contextual factors contributing to the ethics and Corporate Social Responsibility (CSR)-related perceptions, attitudes, and behaviors of young adults. The data collection process has been designed based on lessons learned from previous student surveys on related topics. The reported data have not been used in any of the previous studies.

### Research instrument, constructs, and measures

We used a Web-based questionnaire as our research instrument. It covered multiple research constructs, including individual prosocial attitudes and behaviors, civic and political experiences, and student assessment of their HEIs’ prosocial orientations, including the relevant educational content (related to business ethics, CSR, and environmental sustainability). We used measures that were previously empirically verified, and which proved to be acceptably reliable also in our sample (sufficient level of internal consistency).

We measured *individual prosocial attitudes* by using the individual responsibility subscale of Starett’s^42^ global social responsibility scale, capturing the level of personal caring and responsibility resulting from individual moral judgments. The entire global responsibility scale consists of 62 items, which been validated using the factor analysis with Equimax rotation. However, the scale has not been subjected to convergent or discriminant analysis^57^, which represents a limitation of this empirical study. The same limitation applies to using a subset of items, which has also been the case with psychological research on coping and meaning of life^58^. However, alternative scales were either focused on measuring individual perceptions of CSR^59,60^, or conceptualizing individual social responsibility attitudes by extending the responsible consumer behavior research and using well-known CSR concepts, such as the pyramid of organizational responsibility^61^. Provided that our undergraduate student sample had limited exposure to the corporate sector and a limited experience with the CSR concept, we opted for using the Starett’s subscale, referring to the general social and political issues.

The *individual responsibility subscale* consists of twelve items (e.g., *Helping correct injustice and oppression in the world gives me a feeling of significance* and *A person does not need to worry about other people if only he looks after himself*), of which six are negatively stated and were recoded during the initial data analysis. For each item, the participants rated their agreement on a 9-point scale (1—*completely disagree*, 9—*completely agree*).This subscale provides the benefits of being closely aligned to the concepts of moral judgment and the students’ moral philosophy used in the previous research of related concepts in the SEE region^51^. In addition, it discriminates well among civic-related behaviors, such as volunteering and providing donations for civic causes^42^. In this study, it had somewhat lower internal consistency (⍺ = 0.61), which is still acceptable for exploratory empirical research on non-random samples^62,63^.

*Prosocial behavior* was measured using the Adult prosocialness scale, proposed by Caprara et al.^4^, based on the expressions of empathy and actions of providing help and care to others. Specifically, the scale comprises 16 items tapping into behaviors and feelings about sharing, helping, taking care of, and feeling emphatic with others (e.g., *I help immediately those who are in need, I try to console those who are sad*). It had high internal consistency (⍺ = 0.91). The participants reported their agreement with the items using a 9-point rating scale (1—*completely disagree*, 9—*completely agree*).

*Orientation of higher education institutions* toward the development of prosocial education outcomes was measured using eight items describing the practices of the participants’ HEI (exposure to education content related to business ethics/CSR and environmental sustainability; integrating the topics from these two fields into the core curriculum; encouraging students’ critical thinking toward the educational content; demonstrating a practical commitment to business ethics/CSR and environmental sustainability; bringing in experts and corporate leaders as guest speakers and instructors; cross-sectoral collaboration with corporate, nonprofit and public organizations; using case studies and experiential learning approaches; offering opportunities for service-learning, project-based learning, and internships). The items were adapted from the fourth biennial survey of students studying at business schools participating in the United Nations Principles of Responsible Management Education (PRME) initiative^64:20^. For each item (practice), the participants reported their agreement with a statement (e.g., *At my HEI, all students study environmental sustainability*) using a 9-point scale (1—*not at all*, 9—*completely*). The resulting scale was sufficiently reliable (⍺ = 0.86).

Many *RME areas* and related educational content are taught at different business schools, supporting the UN PRME initiative. We decided to adopt a comprehensive list of twelve RME topics which included CSR; business ethics; ethical decision-making; environmental sustainability; multi-stakeholder management/engagement; diversity management, equal opportunity and non-discrimination; legal aspects of management; responsible consumption and marketing; social entrepreneurship; fair trade and ethical consumption; human rights; anti-corruption; and UN and international organizations/conventions or treaties. The list was adapted from Haski-Leventhal & Manefield’s^64:16^ 2018 report on the student perceptions of RME business school teaching. For each topic, the participants reported how often they heard about them or were taught about them during their studies using a 9-point scale (1—*not at all*, 9—*often*). The scale had high internal consistency (⍺ = 0.95).

## Results

Our sample consisted of 530 undergraduate students enrolled in the business programs at the public universities in Split (Croatia), Sarajevo, and Banja Luka (B&H). The sample structure (41.3% of students were enrolled in Split, 35.7% in Banja Luka, and 23% in Sarajevo) approximately follows the sizes of those three public regional business schools. The mean age of responding students was 23.6 years (with a standard deviation of 5.34 years, since some older students were responding, as well, being enrolled in the business school programs).

The sample is not well balanced in terms of gender since 23.5% of responding students were male and 76.5% female. A similar limitation, however, is present in previous waves of student surveys in the region^1,42^. There seems to be an issue with gender bias in self-administered surveys, regardless of their mode of delivery (paper-based or online), as female respondents tend to respond more often than males^65^. Provided that female business school students tend to place a higher value on moral responsibilities and CSR^66^, there are inherent limitations to obtaining empirical results based on self-administered surveys since the male students seem to assign a lower priority to participate in ethics and CSR-related studies.

While previous research on the nationally representative samples found that, in the SEE region, there might be a lack of social capital and civic initiative outside of the closest social environment^27^, our results show different trends among the business school students. More than half of respondents (51.7%) had volunteer experience in nonprofit or civic settings; 89.9% have donated to nonprofit or public causes; 39.5% were interested in political questions or actively involved in the political process.

To empirically verify the conceptualized model, we have utilized the PROCESS tool^67^, which can reliably assess postulated mediated and moderated relationships. We first created composite variables from the measurement scales using the item-average approach. The construct correlations were then assessed, establishing that low to moderate-level correlations are present. The highest correlation is between higher-educational institution prosocial orientation and RME areas taught (ρ = 0.58, *p* < 0.001).

Since the conceptual model encompasses two parallel mediation streams as well as one moderation and one moderated mediation^68,69^, we have customized one of the pre-programmed PROCESS models (Model 15), programming it only to assess the hypothesized relationships (i.e., the pre-programmed model would also encompass testing the moderating effect of RME areas taught on the personal civic and political involvement relationship with prosocial behavior). The 95% confidence intervals are used on the 5000 bootstrapped samples. The results of the analysis are presented in Table 1.

**Table 1:** Hypotheses tests.

Hypothesis	Hypothesized relationship		Beta (S.E.)	LLCI	ULCI	R^2^
H1a	Individual social responsibility → Personal civic and political involvement		0.21*** (0.04)	0.124	0.302	0.05
H1b	Personal civic and political involvement → Individual prosocial behavior		0.17** (0.05)	0.066	0.272	0.26
H2	Individual social responsibility → Personal civic and political involvement → Individual prosocial behavior		0.04* (0.02)	0.011	0.067	
H3a	Individual social responsibility → HEI social orientation		0.18** (0.07)	0.047	0.322	0.02
H3b	HEI social orientation → Individual prosocial behavior		− 0.07^NS^ (0.08)	− 0.232	0.087	0.26
H4	Individual social responsibility → HEI social orientation*RME areas taught → Individual prosocial behavior	RME areas taught—low	0.01^NS^ (0.01)	− 0.011	0.037	
		RME areas taught—medium	0.03** (0.02)	0.004	0.061	
		RME areas taught—high	0.04** (0.06)	0.006	0.083	
H5a	Individual social responsibility*RME areas taught → Individual prosocial behavior		0.04** (0.02)	0.011	0.068	
H5b	HEI social orientation*RME areas taught → Individual prosocial behavior		0.05** (0.02)	− 0.001	0.092	
H6	RME areas taught → Individual prosocial behavior		− 0.46*** (0.16)	− 0.764	− 0.153	

*NS* not significant, *S.E.* standard error, *LLCI* lower level confidence interval, *ULCI* upper level confidence interval.

*p < 0.1, **p < 0.05, ***p < 0.001.

The results show that individual social responsibility positively and significantly impacts HEI social orientation (β = 0.18, *p* < 0.05) and personal civic and political involvement (β = 0.21, *p* < 0.001), which confirms our hypotheses H1a and H3a. Although there is a significant relationship between individual social responsibility and posed outcomes, such a relationship has a small effect size, with R^2^ values being 0.02 and 0.05, respectively, demonstrating there are other determinants of HEI social orientation and personal civic and political involvement, which are unobserved in this model.

Personal civic and political involvement is positively and significantly related to prosocial behavior (β = 0.17, *p* < 0.05), supporting hypothesis H1b. Simultaneously, HEI social orientation does not have a significant direct link to prosocial behavior, not supporting hypothesis H3b (β =  − 0.07, *p* > 0.05). In terms of the indirect effect of individual social responsibility on individual prosocial behavior through personal civic and political involvement, a significant indirect effect (β = 0.04, *p* < 0.1) supports the H2.

When we test the interaction (moderating effect) of RME areas taught, we observe some unexpected findings. Primarily, the RME areas taught have a strong, direct, and significant negative effect on prosocial behavior (β =  − 0.46, *p* < 0.001), showing that students do not perceive that such education benefits them when the HEI social orientation is not present, or not considered. This finding offers no support for our hypothesis H6. On the contrary, it shows that the effect is opposite than expected.

However, in its moderator role, this model component outlines its ‘booster effect’ for the focal concepts. Namely, with HEI social orientation, which appears not to have a significant direct effect on prosocial behavior (as outlined above with the H3b), the significant moderating effect of RME areas taught (β = 0.05, *p* < 0.05) shows that the more frequently RME areas are taught at a business school, the more potent the effect of the HEI social orientation on prosocial behavior is, which supports hypothesis H5b. Furthermore, we found a significant indirect effect of individual social responsibility on prosocial behavior through HEI social orientation with the moderator present. Namely, the main indirect effect stays insignificant at the low level of RME areas taught. In contrast, at the medium and high level of RME areas taught, the effect of HEI social orientation becomes positive and significant (medium-level RME (value = 5.58) assumes the indirect effect of 0.03 [0.004, 0.063] and high-level RME (value = 7.17) assumes the indirect effect of individual social responsibility of 0.04 [0.006, 0.085], supporting hypothesis H4 as well as H5b. We further demonstrate the significance of our moderated mediation model by observing the index of moderated mediation^56^, with an index value of 0.02 and the confidence interval from 0.001 to 0.018.

Finally, we also confirm the positive moderating effect of RME areas taught on the direct relationship between individual social responsibility and prosocial behavior (β = 0.04, *p* < 0.05), which further confirms that RME areas taught boost the effect individual social responsibility has on prosocial behavior, consequently confirming the hypothesis H5a. Regarding individual prosocial behavior, we capture a medium-size effect^70^, explaining 26% of the variance in the observed model.

## Discussion

While generational and regional factors matter, recent research has shown that individual factors are essential for converting attitudes into appropriate behaviors. Kadić Maglajić et al.^71^ demonstrate that the individual levels of prosocial and pro-environmental orientation predict socially responsible consumption, with emotional intelligence boosting both effects. Along with the responsible consumption choices, consideration of organizational CSR when seeking employment is often singled out as a significant determinant of young adults’ attitudes and their conversion into socially responsible behavior^48^. In the non-Anglo-Saxon context, Klimkiewicz and Oltra^72^ empirically verify that the CSR-based employment intentions of Polish studies depend on their interpretations of the social responsibility construct. These arguments support an essential role of individual perceptions and interpretations of organizational CSR, which should also be considered in higher education. They are also shown to be relevant for business schools and their RME initiatives by our empirical results on the RME teaching and learning areas, having a mixed influence on students’ prosocial behavior.

While the direct negative influence of RME areas on prosocial behavior could be interpreted in terms of students’ low understanding of RME (or the motivation for its acceptance), at first sight, the role of RME areas in the moderated mediation model seems to be puzzling. But the finding that coupling RME teaching and learning areas with the HEI prosocial orientation leads to synergetic effects in developing students’ prosocial behavior is entirely consistent with previous research in the SEE region that analyzed the role of students’ mental gap between what their HEIs should do and what they are doing in terms of CSR^1^. On the other hand, the fundamental finding of this study is related to the relativization of CSR practices, which students feel to be imposed on them when there is no evidence of ‘walking the talk’ (i.e., HEIs adopting the prosocial orientation in practice). At least in the SEE region, these results show that professional socialization is much less at work in HEIs than in the students’ feelings of authenticity and civic and political involvement in constructing their perceptions of how their schools’ CSR is to be created and practiced.

The topics of cynicism, disillusionment, and system delegitimation are often found in post-communist and post-transitional countries^73,74^. Even in the undergraduate student body, they seem to work as a “default setting.” However, the data on young adults’ civic and political involvement suggest that new student generations are increasingly more active and involved. Nevertheless, some of the discussed characteristics could be due to generational differences, although the specific regional and cultural differences are still at work^75^.

Namely, even for the preceding generation of ‘Millenials’ (i.e., the ‘Generation Y,’ born in the 1980–1995 period), it is reported that perceived authenticity is essential for their interpretation of organizational CSR. Based on qualitative research, Chatzopoulou and de Kiewiet^76^ report that natural skepticism and idealistic moral philosophy motivate young adults to question the motives behind organizational CSR initiatives and proclamations. They value the consistent commitment to CSR rather than one-time philanthropic initiatives, or the executives’ proclamations^77^, especially when they are used to address the threats to organizational reputation^78^. It is encouraging that, at least in the post-transition countries, such as the Czech Republic, younger generations are more accepting of CSR^79^ within the previously described limitations.

Our findings related to the ‘booster effect’ of the RME areas on HEI social orientation as a predictor of students’ prosocial behavior, confirm the relevance of RME teaching and learning in business schools^48^. This shows that, even in the regions that could be considered peripheral, HEIs can achieve a positive social impact when aligning their organizational practices with pronounced strategies and values. This is supported by previous research, suggesting that the ‘copy-and-paste’ approach, based on the local, or regional implementation of what is considered the ‘world class’ CSR in higher education, might not work^80^. Along with understanding and accounting for the effect of many different generational or regional factors, our research results show that the same level of perceived HEI commitment could be the key to achieving the success of RME in business schools.

While the low number of RME areas taught does not provide a synergetic effect on the HEI social orientation, the higher number of RME teaching and learning areas adopted provide a high level of interaction with the HEI social orientation and create a higher social impact. Therefore, we propose that the level of commitment shown by an HEI, in terms of socially responsible academic teaching and learning and HEI’s organizational practices, could be the fundamental factor in predicting their social impact.

It is also difficult to comment on how HEIs might influence the potential students’ social experiences and engagement in their civic and political initiatives. Although our empirical results reveal some promising insights, this issue deserves a separate study. In SEE, regional studies of social capital, trust, and prosocial behavior show a generally low level of trust in public institutions, with a focus on friends, family, and other selected individuals, instead of developing more inclusive social initiatives, public policies, and responses to crises^27,81–84^. In the post-socialist and other peripheral regions, there are embedded social elites’ beliefs that the other parts of the society cannot be trusted, leading to high levels of informal behaviors^85^, including governance practices. When followed by the interplay of conformity, fatalism, and high levels of social frustration among the underprivileged social groups, a vicious circle forms, leading to particularism and low civic or political activism^86^.

In our sample of business school students, individual social responsibility, when moderated by the RME areas taught, had a direct and significant relationship with prosocial behavior, regardless of the level (low, moderate, or high) of RME content, to which the students have been exposed. This shows that, even for the factors unobserved by this model, business schools can positively influence the conversion of young adults’ attitudes into behaviors.

Our empirical analysis of the second mediation stream shows a positive and statistically significant relationship between young adults’ social responsibility and prosocial behavior, mediated by their civic and political involvement. As indicated by the previous discussion, such an empirical result would be unexpected, to a high degree, provided that the regional and generational factors should inhibit young adults’ civic and political activism and contribute to their cynicism and disillusionment. Nevertheless, there could be a positive and potentially generalizable HEI influence on this relationship, which needs to be confirmed by future research.

## Conclusion, research limitations, and future empirical research

Our study conceptualized the relationships between individual-related characteristics and HEI-related orientations, which focus on the prosocial aspect of sustainability and how those behaviors and activities shape individual prosocial behavior. We assess the effect of individual social responsibility through parallel mediations. Firstly, through personal involvement in civic and political activities, and secondly, through the HEI social orientation as a driver. Furthermore, we assess the RME areas taught at the HEI as a potential moderator of the latter mediation and the direct individual social responsibility effect. Findings are summarized in a hypothesis overview in Table 2.

**Table 2:** Hypotheses overview.

Hypothesis	Hypothesized relationship	Research results
H1a	Individual social responsibility → Personal civic and political involvement	Supported
H1b	Personal civic and political involvement → Individual prosocial behavior	Supported
H2	Individual social responsibility → Personal civic and political involvement → Individual prosocial behavior	Supported
H3a	Individual social responsibility → HEI social orientation	Supported
H3b	HEI social orientation → Individual prosocial behavior	Not supported
H4	Individual social responsibility → HEI social orientation*RME areas taught → Individual prosocial behavior	Supported
H5a	Individual social responsibility*RME areas taught → Individual prosocial behavior	Supported
H5b	HEI social orientation*RME areas taught → Individual prosocial behavior	Supported
H6	RME areas taught → Individual prosocial behavior	Not supported

Here, we show that most of the hypothesized relationships are confirmed, but we also see some unexpected findings. Firstly, the apparent effect of HEI social orientation on individual prosocial behavior is insignificant, and we cannot assess the relationship. When we account for the impact of the RME areas taught at the HEI, however, we see that the larger the scope of RME areas taught, the stronger the effect of individual social responsibility through HEI social orientation is (the impact turns from an insignificant to a significant one). Furthermore, we find it interesting that the direct effect of RME areas taught is negative, i.e., the more intensive the teaching plan is—the more reluctant the students seem to be to engage in individual prosocial behavior. This shows that the RME areas taught need to be coupled with specific concrete actions demonstrated through HEI social orientation.

This study is not without limitations. The sample is restricted to three mid-sized business schools only. While those are located in culturally similar areas of South East Europe, this area is considered a peripheral European region with mixed experiences of transition, modernization, and ‘Europeanization’ practices^87,88^, which makes the results hard to generalize. The findings should thus be re-evaluated in different socio-cultural environments, as implied by a call for social psychology to engage in the study of prosocial behaviors’ socio-cultural and ideological contexts^89^.

The cross-sectional research design is another limitation of the study, for it offers no information on the causality or directionality of the observed relationships. Moreover, it implicitly assumes the relationships between the individual social responsibility, prosocial behavior, and the HEI social orientation are invariable over time and across different HEI environments. This is not entirely realistic, as business school students might have vastly different value profiles^90^ and professionalization/socialization patterns^6^ than students of technical, social sciences, and humanities. The research timing could entail implications for the generalizability of empirical findings due to the influence of the global coronavirus pandemic on the levels and patterns of prosocial behaviors^91^, especially among young adults^92^.

Future empirical research on this topic would benefit from longitudinal and comparative research designs, acknowledging the influence of different academic and social contexts on the discussed constructs. In terms of various contexts and behaviours, it is also of note that our measure of prosocial behaviors was unidimensional and did not account for different forms of behaviors. While some (regional) authors posit that different forms of civic engagement should not be considered independent^93–95^, and the scale used comprises different forms of prosocial behavior (see Research instrument, constructs, and measures), the specific context of business schools and higher education might be more conducive in promoting certain types (or specific) activities. Finally, future research should also address the gender imbalance in the sampling to account for its potential influence on the findings. In addition to self-selection and differences in attitudes (discussed in Results), the possible influence might also be attributed to the gender differences in work ethics and grades, which seem to be relevant even in the most gender-equal social environments^96^. This is not an easy feat, however, as gender imbalance is found often in research samples, especially if there is a single wave of data collection^97^.

## Supplementary Information

Supplementary Information.

## Data Availability

Data generated or analyzed during this study are included in this published article (and its Supplementary Information files).
